# Antenatal Seroprevalence of Zika and Chikungunya Viruses, Kingston Metropolitan Area, Jamaica, 2017–2019

**DOI:** 10.3201/eid2802.211849

**Published:** 2022-02

**Authors:** Joshua J. Anzinger, Chadwic D. Mears, A.E. Ades, Keisha Francis, Yakima Phillips, Ynolde E. Leys, Moira J. Spyer, David Brown, Ana M. Bispo de Filippis, Eleni Nastouli, Thomas Byrne, Heather Bailey, Paulette Palmer, Lenroy Bryan, Karen Webster-Kerr, Carlo Giaquinto, Claire Thorne, Celia D.C. Christie, on behalf of the ZIKAction Consortium,

**Affiliations:** The Global Virus Network Jamaica Affiliate, Kingston, Jamaica (J.J. Anzinger);; The University of the West Indies, Kingston (J.J. Anzinger, C.D. Mears, K. Francis, Y. Phillips, Y.E. Leys, P. Palmer, L. Bryan, C.D.C. Christie);; University of Bristol, Bristol, UK (A.E. Ades);; University College London, London, UK (M.J. Spyer, E. Nastouli, T. Byrne, H. Bailey, C. Thorne);; Instituto Oswaldo Cruz, Rio De Janeiro, Brazil (D. Brown, A.M.B. de Filippis);; University College London Hospitals NHS Trust, London (E. Nastouli);; Ministry of Health, Kingston (K. Webster-Kerr); University of Padova, Padua, Italy (C. Giaquinto)

**Keywords:** Zika virus, chikungunya virus, prevalence, antenatal, vector-borne infections, Kingston Metropolitan Area, Jamaica, Caribbean, viruses

## Abstract

To determine the extent of exposure to Zika virus (ZIKV) and chikungunya virus (CHIKV) in Jamaica, we collected serum from 584 pregnant women during 2017–2019. We found that 15.6% had antibodies against ZIKV and 83.6% against CHIKV. These results indicate potential recirculation of ZIKV but not CHIKV in the near future.

The recent introductions of chikungunya virus (CHIKV) and Zika virus (ZIKV) in the Americas led to widespread epidemics with substantial health and economic effects. Small island developing states in the Caribbean are particularly affected by emerging mosquitoborne disease, primarily because of year-round climatic conditions favorable for mosquito breeding, high levels of poverty, and extensive migration.

In Jamaica, introductions of CHIKV in 2014 and ZIKV in 2016 led to epidemics that overwhelmed the healthcare system (*1*,*2*). Despite the Caribbean being greatly affected by the CHIKV and ZIKV epidemics, information regarding the extent of population exposure is limited; most previous studies examined high-resource Caribbean islands. Studies performed after the introduction of CHIKV into the Caribbean showed CHIKV seroprevalence to be Guadeloupe 48.1% (*3*), Haiti 78.7% (*4*), Martinique 41.9% (*3*), Puerto Rico 23.5% (*5*), and Saint Martin 16.9% (*6*). A serosurvey of participants in rural and urban areas in Suriname in 2017 showed a ZIKV seroprevalence of 35.1% overall and 24.5% in a remote village (*7*); in Martinique in 2016, seroprevalence of blood donors was 42.2% (*8*).

To determine seroprevalence in the greater Kingston, St. Andrew, and St. Catherine metropolitan region in Jamaica, we performed a CHIKV IgG and ZIKV IgG serosurvey of 584 pregnant women attending 5 public antenatal clinics in the Kingston Metropolitan Area (KMA) from June 28, 2017, through April 15, 2019. Pregnant women >16 years of age attending 1 of the 5 antenatal clinics and planning to deliver at 1 of the 3 KMA public maternity hospitals were eligible for enrollment. The South East Regional Health Authority report of 20,817 total live births at the 3 KMA public maternity hospitals from July 2017 through April 2019 indicates that our study represents ≈3% of pregnant women delivering at a KMA public maternity hospital. 

To detect antibodies for each virus, we used the Euroimmun chikungunya virus IgG ELISA (https://www.euroimmun.com) and BLACKBOX Zika virus ELISA (Bernhard-Nocht-Institut für Tropenmedizin, https://www.bnitm.de). Because serologic cross-reactivity with related flaviviruses can be problematic for accurately identifying past ZIKV infections, it is imperative that ZIKV serologic assays account for cross-reactive antibodies. The BLACKBOX Zika virus ELISA is a ZIKV immune complex–binding IgG ELISA that is highly specific and does not show cross-reactivity with dengue virus (DENV) (*9*). For prevalence estimates, we calculated Clopper-Pearson CIs. This study was approved by the University of the West Indies Ethics (ECP 100 18/19) and Ministry of Health Ethics Committees (2017/06).

Among women attending all antenatal clinics, 83.6% (95% CI 80.0%–86.5%, range 72.2%–88.1%) were positive for CHIKV IgG (Table), and 15.6% (95% CI 12.7%–18.8%, range 12.6%–22.4%) of samples tested were positive for ZIKV (Table). Of the 91 ZIKV IgG–positive women, 72 were also positive for CHIKV IgG, indicating a highly significant odds ratio for the association (21.9, 95% CI 13.8–36.7), probably resulting at least in part from the 2 viruses being transmitted primarily by *Aedes aegypti* mosquitoes. 

**Table:** Chikungunya and Zika virus results for 584 pregnant women attending 5 public antenatal clinics, Kingston Metropolitan Area, Jamaica, June 28, 2017–April 15, 2019

Virus, clinic	Tested, no.	Result			
		Negative, no.	Equivocal, no.	Positive, no. (%)	
Chikungunya*					
A	54	11	4	39 (72.2)	
B	159	19	0	140 (88.1)	
C	76	8	2	66 (86.8)	
D	197	34	5	158 (80.2)	
E	98	9	4	85 (86.7)	
Total	584	81	15	488 (83.6)	
Zika†					
A	54	44	0	10 (18.5)	
B	159	138	1	20 (12.6)	
C	76	64	1	11 (14.5)	
D	197	166	3	28 (14.2)	
E	98	75	1	22 (22.4)	
Total	584	487	6	91 (15.6)	

*Euroimmun CHIKV IgG ELISA (https://www.euroimmun.com). †BLACKBOX ZIKV IgG ELISA (https://www.bnitm.de).

To ensure that the seroprevalence results were not inflated by false positives, we also examined 89 archived serum samples from pregnant women who had attended 1 of the 5 clinics examined (clinic D) during June–December 2013. This period predates CHIKV and ZIKV introductions into Jamaica and was a period of low DENV circulation, similar to 2017–2019 (Ministry of Health Epidemiology Bulletin, https://www.moh.gov). All 89 samples were negative for CHIKV IgG and ZIKV IgG (no results were equivocal), indicating that false-positive results were unlikely to affect the reported CHIKV and ZIKV seroprevalence for 2017–2019.

Our study indicates a high level of past CHIKV infections and a low level of ZIKV exposure among pregnant women in Jamaica receiving antenatal care during 2017–2019. We offer several possible explanations for the higher seroprevalence of CHIKV compared with ZIKV. CHIKV infections result in substantially greater viremia, which could lead to increased transmission rates for CHIKV. In addition to the lower viremia associated with ZIKV, preexisting antibodies to DENV can be cross-protective against ZIKV infection (*10*), which may have limited transmission in Jamaica. During the 2016 ZIKV epidemic in Jamaica, wide circulation of DENV (Appendix Figure) could have limited ZIKV transmission because of cross-reactive antibodies generated during DENV infection or competition between the 2 viruses during cocirculation.

Cases of CHIKV and ZIKV infection in Jamaica have been extremely limited since their initial epidemic years. Only 21 CHIKV cases were reported in Jamaica during 2015–June 2020, and only 1 ZIKV case was reported during 2017–June 2020. CHIKV cases will probably remain limited in Jamaica until a more substantial portion of the population lacks immunity. In contrast, ZIKV could possibly circulate again in Jamaica in the near future because of low population immunity and waning cross-reactive DENV antibodies.

Appendix Additional methods and results for study of antenatal seroprevalence of Zika and chikungunya viruses, Jamaica, 2017–2019.
