# Supplementary material for: Antenatal Seroprevalence of Zika and Chikungunya Viruses, Kingston Metropolitan Area, Jamaica, 2017–2019
Source: Emerg Infect Dis. 2022 Feb;28(2):473–5. doi: 10.3201/eid2802.211849 (PMC8798668; doi:10.3201/eid2802.211849)
Supplement: Appendix — Additional methods and results for study of antenatal seroprevalence of Zika and chikungunya viruses, Jamaica, 2017–2019. [file 21-1849-Techapp-s1.pdf]

Article DOI: https://doi.org/10.3201/eid2802.211849

Antenatal Seroprevalence of Zika and Chikungunya Viruses, Kingston Metropolitan Area, Jamaica, 2017–2019

Appendix

Methods

Patients meeting the Jamaican Ministry of Health 2016 case definition for ZIKV or DENV and with a serum sample collected <6 days after symptom onset were tested by ZIKV real-time reverse transcription PCR (RT-PCR) (by using the method of Lanciotti et al., [*1*]) and DENV NS1 (SD. Bioline). ZIKV real-time RT-PCR was also performed for Zika patient urine samples patients collected up to 14 days after symptom onset. The 2016 case definition for ZIKV was a patient with rash or elevated body temperature (>37.2°C) with ≥1 of the following: arthralgia or myalgia, headache or malaise, nonpurulent conjunctivitis or conjunctival hyperemia. The 2016 case definition for DENV was a patient with an acute febrile illness (>37.2°C) of 2–7 days duration with ≥2 of the following: headache, retro-orbital pain, myalgia, arthralgia, rash, hemorrhagic manifestations, leukopenia. All pregnant women with a rash only and other symptoms met the definition of having a suspected case for both ZIKV and DENV. For serum samples tested for both ZIKV real-time RT-PCR and DENV NS1, 148 samples tested ZIKV real-time RT-PCR positive with only 6 samples testing positive for both ZIKV RT-PCR and DENV NS1, indicating an at least 95.9% specificity in the context of ZIKV for the SD. Bioline DENV NS1 ELISA (Appendix Figure).

Reference

1. Lanciotti RS, Kosoy OL, Laven JJ, Velez JO, Lambert AJ, Johnson AJ, et al. Genetic and serologic properties of Zika virus associated with an epidemic, Yap State, Micronesia, 2007. Emerg Infect Dis. 2008;14:1232–9. PubMed https://doi.org/10.3201/eid1408.080287

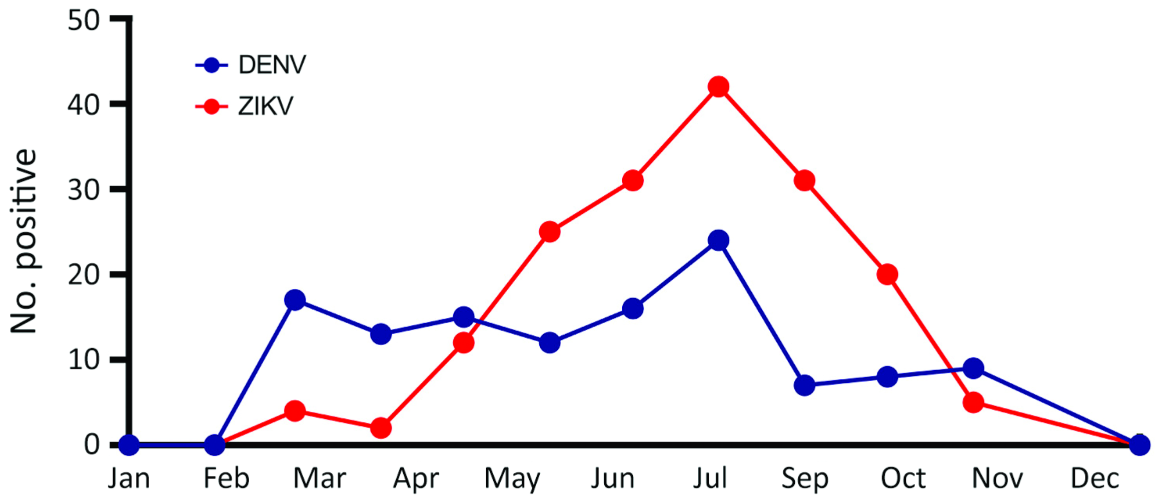

Appendix Figure. Zika virus (ZIKV) and dengue virus (DENV) cases with laboratory evidence of acute infection, Jamaica, 2016.
